# Effect of Restricted Group Sandplay on Interpersonal Sensitivity in College Students

**DOI:** 10.3389/fpsyg.2021.771209

**Published:** 2021-12-17

**Authors:** Shuhan Yu, Liyu Zhan

**Affiliations:** ^1^Department of Economics and Management, Fujian Chuanzheng Communications College, Fuzhou, China; ^2^College of Forestry, Fujian Agriculture and Forestry University, Fuzhou, China

**Keywords:** group sandplay, sandplay feature coding, COVID-19, college student, interpersonal sensitivity

## Abstract

This study aims to investigate the intervention effect of group sandplay on the interpersonal sensitivity of college students and analyze the relationship between the theme and interactive behavior characteristics and the intervention effect of group sandplay especially during the period of COVID-19. Sixty college students were randomly assigned to the experimental group (group sandplay) or the control group (neutral task interventions). The results showed that the interpersonal sensitivity level of the experimental group was significantly lower than that of the control group. For the experimental group, the variation in the interpersonal sensitivity level was significantly negatively correlated with the change in warm, supportive behavior during group sandplay interaction. These findings suggest that group sandplay is effective in improving the interpersonal sensitivity level of college students, and this effect can be positively predicted by warm and supportive interaction behaviors in group sandplay.

## Introduction

Interpersonal sensitivity is a common source of psychological distress in college students (Sfendla and Hadrya, 2020). Interpersonal sensitivity is an integrated personality trait characterized by excessive vigilance and sensitivity to others’ emotions, affect, behaviors, and cognition; suspicion and fear of “criticism and rejection”; accompanying social avoidance and separation anxiety; and ongoing concern about social threat (You et al., 2019). Studies have shown that interpersonal sensitivity is a psychological risk factor for infectious and cardiovascular disease and may increase the risk of depression, anxiety, and aggressive behavior (Kay et al., 2004; Denollet, 2013). Therefore, effective reduction of an individual’s interpersonal sensitivity trait or tendency is important to his or her physical and psychological health. Group psychological counseling has been widely used in the study of interpersonal communication intervention of college students, however, different from group psychological counseling, which works on the level of consciousness, group sandplay therapy makes full use of non-verbal communication and symbolic meaning to work on the level of unconsciousness (Roesler, 2019). Previous studies have shown that group sandplay therapy can significantly improve individuals’ cognitive empathy, behavioral empathy, self-expression, and appropriate rejection skills and significantly decrease interpersonal sensitivity in college students with relatively poor social and empathic skills (Zhang et al., 2010). For example, in Zhu et al. (2015) study, 30 college students whose interpersonal skills score and empathy score are both lower than overall’s (*n* = 241) 40% were selected and randomly divided them into sandplay group and counseling group to take 6 weeks’ group sandplay and counseling intervention, results showed that post and after 2 months in cognitive empathy, acts empathy, appropriate rejection and self-expression were higher than before, while the scores which post in interpersonal sensitivity was less than before in sandplay group. A group sandplay therapy combines the advantages of both sandplay therapy and group therapy: sandplay therapy is an unconscious, self-healing process and group therapy enables participants to build trust and respect toward others and to become aware of diverse viewpoints (Sweeney and Homeyer, 1999). Group sandplay therapy can also significantly decrease tendencies toward social avoidance and painful feelings and can improve social awareness and coordination skills in college students with social anxiety (Zhang et al., 2011). Regarding the causes underlying the intervention effect of sandplay, first, sandplay promotes the healing function of self-exploration in participants. According to Dora Kalff, the inventor of sandplay therapy, the separation of consciousness and unconsciousness leads to disintegration of the self, which in turn induces psychological conflict. Sandplay serves as an effective medium for the transition from unconsciousness to consciousness, thereby helping individuals to better understand and accept themselves. Second, in group sandplay, the sandplay field can be used to bridge psychological activities at the unconscious and conscious levels, and the individual’s invisible inner world can be externalized as a visible symbolic representation during sandplay, thereby facilitating emotional catharsis, self-examination and regulation. Lastly, the theory of group dynamics explores the generation mechanism of group behavior with the help of “field” in physics, and holds that it is easier to change individuals by influencing the group than by directly changing individual members to form a complex mutual relationship among people in a group; the interaction between a group and its individual members creates a group dynamic, cohesion, driving force and dissipation force influence and balance each other, forming a joint force to determine the direction of group development (Lewin, 1948). Based on the group dynamics theory, group sandplay creates an accepting, inclusive, and supportive atmosphere and takes advantage of group dynamics to stimulate cooperative consultation and empathic understanding among the group members, thereby enabling them to acquire more interpersonal skills and apply them in real life. As a common mode of group sandplay, restricted group sandplay has restricted specifications and a behavioral code^1^ that objectively encourages participants to define their own social roles, master the skills to establish and maintain interpersonal relationships, enhances team spirit and awareness of rules, and ultimately achieves self and team growth.

Since the outbreak of the Coronavirus disease 2019 (COVID-19) at the end of 2019, a series of effective epidemic preventive and control strategies have been developed and conducted by the Chinese government to curb the spread of the virus. The general populations, especially the young people, are suggested to stay at home in quarantine. Although many studies have provided experimental evidence that group sandplay improves the quality of social relationships, interpersonal trust, interpersonal skills, and interpersonal distress of college students, most research has focused on populations with obvious social anxiety and disorders and has somewhat neglected the effect of sandplay intervention on the interpersonal sensitivity of average populations. In fact, past empirical studies of group sandplay therapy paid less attention to the impact of group sandplay on the interpersonal skills of ordinary college students. In addition, few studies have addressed the relationship between the features and the effect of sandplay, in particular, the internal relationship between the quantified sandplay behavior and psychological indicators have hardly been paid attention to. Therefore, this study used restricted group sandplay as the intervention paradigm and a general college student population as the study objects. The study focused on the interpersonal sensitivity level of the experimental group and the control group. The experimental group underwent the sandplay intervention, and the control group underwent a neutral task. The sandplay work coding form to code information about the themes and interactive behaviors of the sandplay work were used to quantitatively analyze the intervention effects and characteristics of the sandplay work. Because of above-mentioned views, the main hypothesis of this study is that, compared with neutral intervention, after sandplay treatment, the interpersonal sensitivity of college students would be significantly reduced, and the supportive behaviors such as “empathic cooperation,” “participation and construction” and the healing characteristics of the sandplay would be significantly improved, while the destructive behaviors of “Entering another participant’s domain” and traumatic characteristics of the sandplay would be significantly reduced.

## Materials and Methods

### Participants

Sixty college students with a mean age of 18 years (age: *SD* = 0.184; gender: *M* = 0.5, *SD* = 0.504) were recruited from Fuzhou City, Fujian Province, China during 2019–2020. They were randomly assigned to one of two groups: An experimental group (females: 15, males:15) and a control group (females: 15, males:15). All of the students were physically healthy. They were examined using the Symptom Checklist 90 (SCL-90) and showed no psychological or psychiatric disorder. None of them reported severe social anxiety. For the SCL-90 scale, the homogeneity reliability of total scale is 0.97, the homogeneity reliability of each subscale is above 0 69, the retest reliability is greater than 0 7, and the content validity and structure validity are good. Informed consent was signed by all participants, who were rewarded with a report on their psychological health based on testing with the SCL-90 psychometric instrument and an in-depth discussion of the test report with a psychological consultant after they completed the experiment. The study was approved by Institutional Review Board (IRB) of College of Forestry in Fujian Agriculture and Forestry University of China.

### Experimental Procedure

#### Group Sandplay and Neutral Interventions

The experiment was conducted by referencing and modifying the restricted group sandplay therapy method of Zhang et al. (2011). None of the subjects were informed of the purpose of the experiment. The experimental and control groups were each divided into six teams (five people per team) and underwent 3 weeks (one session each week, 55–60 min per session) of group sandplay and neutral interventions, respectively. The group sandplay sessions were conducted by two psychological consultants (experimenters) who organized the sessions, recorded the subjects’ behaviors throughout the sessions (see the “Operational definition” in Table 1. Interaction behavior coding of sandplay), and ensured the implementation of the predetermined rules, namely, that only one action was allowed per person per turn, no communication of any form was allowed during sandplay, and the person who took the last action in a sandplay work was allowed to make final adjustments to the work. The time of each turn ends when the last subject is placed, the whole sandtable activity lasts about 55–60 min. After a sandplay work was completed, the experimenters led the participants in sharing the intents of their sand tool placements and their feelings about the other participants’ sand tool placements and in describing the theme of the sandplay through group consultation. After a sandplay session was completed, the experimenters asked the participants to decide whether to retain or dismantle the work. The sandplay work could be dismantled by the participants or by the experimenters after the participants left. The basic procedure of the neutral intervention was as follows. The participants were asked to recall their diets and schedules in the previous 7 days and write them down in as much detail as possible. After the experiment was completed, the participants were allowed to take away the written materials. The participants gave informed consent to this study, voluntarily cooperated in the evaluation, and signed the informed consent form. Written informed consent was obtained before the experiments, and the study was approved by the committee of ethnic board of Fujian Agriculture and Forestry University and the latest revision of the Declaration of Helsinki.

**TABLE 1:** Interaction behavior coding of sandplay.

Code name	Code meaning	Operational definition
Empathic response	Empathic companionship and response.	Responding empathetically to other people’s sand tools with sand tools.
		Actively feeling and responding to the needs of other participants.
Participation in co-construction	Supporting and helping others, expressing care, and sharing one’s own things.	Placing a caring sand tool; symbolically feeding or caring for others; sharing things with peers.
Entering another participant’s domain	Actions of entering another participant’s domain.	Placing a sand tool needed by oneself into the space created by another participant.
Warm support	Actions that support others.	Expressing warmth toward another participant by placing sand tools or manipulating the sand.
Moving a sand tool placed by another participant	Moving.	Moving a sand tool.
Destructive	Interfering or disturbing actions.	Throwing sand or sand tools at another participant; fighting or readiness to fight.
		Actions that disturb other participants playing the game.

#### Sandplay Theme and Interaction Behavior Coding

Based on the *Table of Individual Sandplay Therapy Record* kept during the creation of the sandplay work and the video of the work, the self-compiled sandplay coding manual was used to code six interaction behaviors (empathetic cooperation, participation in co-construction, entering another participant’s domain, warm support, moving a sand tool placed by another participant, and destructive behavior) and the sandplay themes (traumatic and healing). The two experimenters coded the group sandplay video as back-to-back raters, and the rater reliability for both interactive behavior coding and theme coding were greater than 0.85. Whenever a feature that matched the coding table (Tables 1, 2) appeared in the sandplay work, a score of 1 was recorded, regardless of the number of occurrences; if a feature in the coding table did not appear in the sandplay work, a score of 0 was recorded.

**TABLE 2:** Theme coding of sandplay.

Code name	Code meaning	Operational definition
Traumatic theme	Confusion	e.g., The sand implements of different species are scattered in the sand tray, and there is no connection between the sand implements
	Inane	e.g., More than a quarter of the surface product of the sand plate is empty
	Restrictive	e.g., Animals, people are trapped in a square, and the outer boundary ditch is not good
	Neglect	e.g., The client is at a great distance from the subject
	Conceal	e.g., Dangerous sand tools are hidden behind other sand tools
	Lean	e.g., Sand oblique
	Injured	e.g., Animal or human being bitten
	Blocked	e.g., A person or an animal with a bar or other hindrance in front of it cannot move forward
	Inversion	e.g., The sand gear is turned upside down and placed in the sand tray
	Incomplete	e.g., There are no human or movable objects in the real scene
	Trap	e.g., The feet of people and animals sink into the sand more than 30%
	Attack	e.g., An attack between two animals or two higher animals
	Else	e.g., Hungry people and animals are looking for food
Healing theme	Link	e.g., There is a joint of structures such as Bridges between the two partitioned parts
	Journey	e.g., The aircraft has enough runway to take off; The ship sailing; The bus has a way out
	Energy	e.g., Out of the green color of the plant, green continent
	The new born	e.g., The birth of a baby
	Variety	e.g., Creatively use sand tools or sand
	Central tendency	e.g., In the middle of the sand dish is a “round” “organization”
	Integrated	e.g., The main topic or story of the sand dish is suddenly unified
	Ritual	e.g., Song and dance to celebrate the ceremony
	Mitigate	e.g., Between the two sides of the battle, there is over five centimeters of sand
	Else	e.g., To find the treasure

#### Interpersonal Sensitivity Assessment

The interpersonal sensitivity of the participants was assessed using the SCL-90 interpersonal sensitivity subscale, which has been widely used in experimental research on interpersonal sensitivity (Bech et al., 2014; You et al., 2019). The subscale consists of nine items (“expecting people around you to be perfect,” “feeling fearful or uneasy when interacting with the opposite sex,” “being prone to emotional hurt,” “feeling that people are unfriendly toward you or do not like you,” “feeling that people do not understand you or sympathize with you,” “feeling uneasy when people look at you or discuss you,” “feeling neurotic about others,” and “feeling uncomfortable when eating in public places”) and uses a five-level scale (0: no; 1: mild; 2: moderate; 3: severe; 4: very severe). The sum of the scores for the nine items was used as the interpersonal sensitivity score. A higher total score indicates more problems with interpersonal interaction, including a sense of inferiority, egocentrism, and negative expectations, while a lower total score indicates better adeptness at interpersonal relations, being confident and self-poised during interpersonal communication, and having positive expectations.

### Experimental Procedure

The experiment used a one-factor between-group design. The between-group variable was the mode of intervention (the group sandplay intervention vs. the neutral intervention). The subjects were assigned to fixed teams for the experiment and underwent 3 weeks of intervention, one session each week. The interpersonal sensitivity and defense mechanisms of the participants were assessed before the first intervention and after the end of the third intervention. For the experimental group, interaction behavior and characteristic theme coding was reevaluated.

## Results

### Analysis of the Characteristics of the Sandplay

A one-way (time: first vs. second vs. last sessions) repeated measures analysis of variance (ANOVA) of the interactive action indexes of the experimental group was performed. The results showed that time had a significant main effect [*F*_(2,28)_ = 6.000, *p* < 0.01, η^2^ = 0.300] on the frequency of empathetic cooperation actions. A Bonferroni *post hoc* test showed that the frequency of empathetic cooperation actions during the last session (*M* = 0.267, *SD* = 0.082) was significantly higher than that during the first session (*M* = 0.000, *SD* = 0.000, *p* < 0.001) and was marginally significantly higher than that during the second session (*M* = 0.033, *SD* = 0.033, *p* = 0.051). For the frequency of participation in co-construction actions, time had a significant main effect [*F*_(2,28)_ = 5.483, *p* < 0.01, η^2^ = 0.159). A Bonferroni *post hoc* test showed that the frequency during the last session (*M* = 2.900, *SD* = 0.305) was significantly higher than that during the first session (*M* = 1.633, *SD* = 0.237, *p* < 0.05). Regarding the frequency of entering another participant’s domain, time had a significant main effect [*F*_(2,28)_ = 3.253, *p* = 0.054, η^2^ = 0.189]. A Bonferroni *post hoc* test showed that the frequency of such actions during the last session was marginally significantly lower than that during the first session.

A one-way (time: first vs. second vs. and last sessions) repeated measures ANOVA of the frequency of healing and traumatic actions was performed. The results showed that time had a significant main effect on the frequency of healing actions [*F*_(2,28)_ = 4.449, *p* < 0.05, η^2^ = 0.241]. A Bonferroni *post hoc* test showed that the frequency of healing during the last session (*M* = 2.733, *SD* = 0.368) was significantly higher than that during the first session (*M* = 1.800, *SD* = 0.182; *p* < 0.05). Regarding the frequency of traumatic actions, time had a marginally significant main effect [*F*_(2,28)_ = 3.215, *p* = 0.055, η^2^ = 0.187]. A Bonferroni *post hoc* test showed that the frequency of traumatic actions during the last session was significantly lower than that during the first session (*p* < 0.05).

### Analysis of Between-Group Difference in Interpersonal Sensitivity Level

**FIGURE 1:**
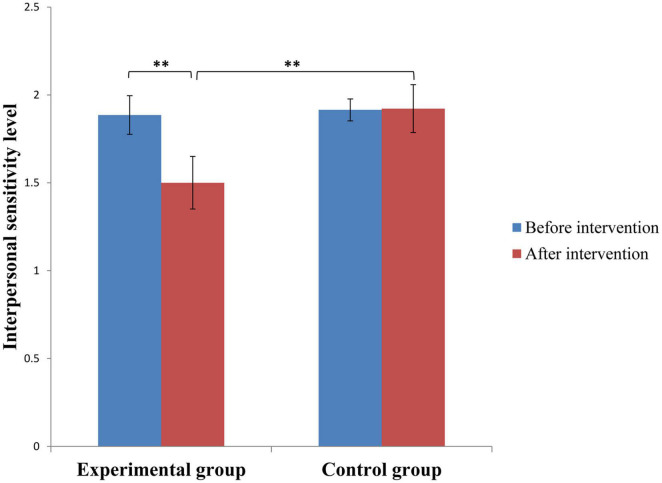
Between-group difference in interpersonal sensitivity level before and after intervention. Error bars represent (–1)/(+ 1)SE; ^**^ means *p* < 0.01.

A 2 (time: before and after intervention) * 2 (groups: experimental and control groups) two-way repeated measures ANOVA of interpersonal sensitivity was performed. The results showed that time had a significant main effect [*F*_(1,58)_ = 4.023, *p* < 0.05, η^2^ = 0.065] and that the interaction between time and group was significant [*F*_(1,57)_ < 1, *p* < 0.05, η^2^ = 0.070]. A simple effect analysis of time was performed. The results show that the difference between the interpersonal sensitivity levels of the experimental and the control groups was non-significant (*F* < 1, *p* > 0.05, η^2^ = 0.000052) before the sandplay or neutral intervention intervention (*M*_*control group*_ = 1.9147, *SD*_*control group*_ = 0.8206; *M*_*sandplay*_ = 1.8857, *SD*_*sandplay*_ = 0.6001) but was significant after the intervention [*F*_(1,58)_ = 6.334, *p* < 0.01, η^2^ = 0.120; *M*_*control group*_ = 1.9220, *SD*_*control group*_ = 0.7454; *M*_*sandplay*_ = 1.5003, *SD*_*sandplay*_ = 0.3409]. More specifically, the interpersonal sensitivity level of the experimental group was significantly lower than that of the control group (*p* < 0.01). A simple effect analysis of the groups was performed. The results showed that the interpersonal sensitivity level of the experimental group after the intervention was significantly lower than that before the intervention (*p* < 0.01), while the interpersonal sensitivity level of the control group after the neutral intervention was not significantly different from that before the intervention (*p* > 0.05) (Figure 1).

### Association Between Interpersonal Sensitivity Level and Frequency of Warm, Supportive Behavior

A correlation analysis of the variation in the interpersonal sensitivity level (difference in measured values before and after the intervention) and variations in the frequency of warm, supportive behavior (the difference between the frequencies during the first and last sessions) in the experimental group was performed. The results showed that the variations in the interpersonal sensitivity level and the frequency of warm support behavior were significantly negatively correlated (*r* = –0.391, *p* < 0.05).

## Discussion

The coding of the features of sandplay showed that the frequency of positive interactive actions (empathetic cooperation and participation in co-construction actions) of the experimental group after the group sandplay intervention was significantly higher than that before the intervention, while the frequency of negative interactive actions (entering another participant’s domain) after the intervention was significantly lower than that before the intervention. In addition, the theme of the sandplay was more healing and less traumatic after the intervention than before the intervention. These results provided a basis for assessing the effect of the group sandplay intervention on improving the interpersonal sensitivity level. Whether group sandplay can effectively reduce interpersonal sensitivity is the main issue addressed in this study. The results show that the interpersonal sensitivity of the experimental group after the sandplay intervention was significantly lower than that of the control group after the neutral intervention and that of the experimental group before the sandplay intervention. The above results indicate that restricted group sandplay helps to improve the personal trait of interpersonal sensitivity in college students.

The internal mediation underlying the effects of group sandplay intervention on interpersonal sensitivity was considered. First, the natural characteristics of sandplay essentially create an inclusive, accepting, and relaxed psychological environment in which individuals can symbolically express their inner world or appropriately release negative emotions and let go of the vigilance, suspicion, or jealousy they may have previously displayed. Second, the rules of restrictive group sandplay objectively constrain individuals and subjectively prevent them from doing whatever they want. This allows them to consciously make cognitive adjustments and behavioral changes during the sandplay activities to dissolve previously held unreasonable beliefs or behavioral styles, which is can help individuals transfer their acquired subjective perceptions and behavioral styles to actual life and learning to appropriately deal with interpersonal confusion, including personal and social confusion and struggles with their relationship with themselves and others. Finally, according to theory of group dynamics, the group dynamics created by group sandplay facilitates individuals’ ability to share their thoughts, ideas, and psychological energy and mutually improve by observing and imitating each other. Vice versa, the resulting fusion among the individuals facilitates the integration and harmonious development of the group. These positive factors equip the group members with more adequate psychological resources for accepting themselves and others, considering things from multiple perspectives, and changing inappropriate beliefs they may have previously held, such as distrust of others and negative thoughts about external judgment. This in turn improves their tendency to be sensitive during interpersonal relationships.

It is worth noting that warm, supportive behavior by individuals during sandplay can predict their interpersonal sensitivity level. In other words, during group sandplay, individuals who display more warm, supportive behavior have a lower interpersonal sensitivity level. Existing research has mainly investigated the relationship between warm support and interpersonal sensitivity in individuals from the perspective of social support. In particular, affective, warm understanding from parents can prevent interpersonal sensitivity, while parenting characterized by excessive intervention or excessive protection is positively correlated with neurotic personality and indirectly affects the interpersonal sensitivity of individuals through neuroticism (Lai et al., 2014; Liu and Wang, 2017). The results of this study suggest that from the perspective of the individual’s own behavior, if an individual shows warm support to others during interpersonal communication, it might have a positive impact on improving his/her interpersonal sensitivity. From the perspective of interpersonal communication, prosocial behavior enhances an individual’s sense of self-esteem, leading to self-satisfaction and promoting interpersonal adaptation (Deborah et al., 2004; Ding and Lu, 2016). Warm, supportive behavior during group sandplay is essentially a kind of prosocial behavior. It increases the closeness of the interpersonal relationship among the group members and reduces vigilance and sensitivity to judgment from others, as well as reducing submissive, inhibitive, and other defensive behaviors.

## Limitations and Outlook

This study has limitations. First, it lacked comprehensive evaluation of the quality of interpersonal relationships and interpersonal skills. The tools used in the literature to assess the interpersonal relationships of individuals include the *Interpersonal Relation Comprehensive Diagnostic Scale*, the *Interpersonal Trust Scale*, the *Simplified Coping Style Questionnaire*, the *Self-Acceptance Scale*, and *Social Anxiety*. This study used only the interpersonal sensitivity subscale of the SCL-90 to assess the effect of the interventions. Therefore, it is necessary to use multidimensional measurement tools to obtain more convincing scientific evidence of the timeliness and durability of the effects of sandplay intervention. Second, this study did not showed that no significant changes of “warm support” and “Moving a sand tool placed by another participant” behavior characteristics were observed after sandplay treatment, however, this does not mean that these two behavioral characteristics are not affected by sandplay activities, it may be related to the insufficient sample size of the subjects, and this result needs to be verified repeatedly by future studies. Third, this study did not provide direct evidence that most of the interactive behaviors of sandplay participants (empathetic cooperation, participation in co-construction, and entering another participant’s domain) and the characteristics of the theme of the sandplay (healing and traumatic) were significantly correlated with interpersonal sensitivity. This may be because the subjects were recruited from a normal population, and the sample size was small. In future research, the sample size will be increased, and the relationship between the features of the sandplay and the intervention effect will be investigated by recruiting subjects with specific personality traits (such as social anxiety). Fourth, this study found that warm support was significantly negatively correlated with interpersonal sensitivity. This finding is partially inconsistent with the findings reported in the literature. Empirical research reveals that individuals with high interpersonal sensitivity or high sensitivity to rejection engage in ingratiating helping behaviors out of motives related to self-interest, such as maintaining relationships, gaining recognition, or avoiding negative evaluations; e.g., in high social stress situations in which most peers help those seeking help, individuals with high interpersonal sensitivity are more willing than individuals with low sensitivity to make more donations (Li and Wang, 2019). Because some individuals undertake prosocial actions for altruistic reasons (such as social accountability and sympathy), and other individuals do so for egoistic reasons (such as seeking rewards and appreciation and increasing self-esteem and self-satisfaction) (Nancy et al., 2016; Sara et al., 2016; Wei et al., 2021), future research will investigate the essential meaning of positive and negative sandplay features from the perspective of the motivation underlying individuals’ altruistic decisions. Finally, interpersonal sensitivity assessment were only test before the first intervention and after the last intervention, in the future studies, it’s necessary to measure the level of interpersonal sensitivity after each intervention to more accurately assess the effects of sandplay therapy.

## Conclusion

This study mainly investigated the intervention effect of restricted group sandplay on interpersonal sensitivity. The variations in interpersonal sensitivity in the experimental group (the group sandplay intervention) and the control group (the neutral intervention) were compared, and the relationship between the interactive actions and the interpersonal sensitivity of the group sandplay participants was analyzed. The results show that restricted group sandplay helps to improve the interpersonal sensitivity of college students. At the same time, warm support for others during interpersonal communication may be of positive significance to an individual’s interpersonal sensitivity.

## Data Availability Statement

The raw data supporting the conclusions of this article will be made available by the authors, without undue reservation.

## Ethics Statement

The studies involving human participants were reviewed and approved by the Institutional Review Board (IRB) of College of Forestry in Fujian Agriculture and Forestry University of China. The patients/participants provided their written informed consent to participate in this study.

## Author Contributions

SY contributed to perform the experiment, collect the data, analyze the results, and wrote the manuscript. LZ contributed to put forward the research ideas, experimental design, and oversee the whole process of research implementation. Both authors have read and approved the final version of the manuscript.

## Conflict of Interest

The authors declare that the research was conducted in the absence of any commercial or financial relationships that could be construed as a potential conflict of interest.

## Publisher’s Note

All claims expressed in this article are solely those of the authors and do not necessarily represent those of their affiliated organizations, or those of the publisher, the editors and the reviewers. Any product that may be evaluated in this article, or claim that may be made by its manufacturer, is not guaranteed or endorsed by the publisher.

## Footnotes

1 **Operation specifications for restricted group sandplay:** (1) Each participant’s turn to place sand tools in the sand tray is determined by drawing lots or playing rock-paper-scissors to ensure that all participants in the group have an equal chance of being the first player. (2) Each participant is allowed to perform only one action at each turn. (3) The participants are not allowed to communicate intentionally using verbal or body language. (4) No participant can remove a sand tool that has already been placed during sandplay, whether by himself or another participant, to outside of sand tray or the toy shelf. However, participants are allowed to move sand tools as an action. After moving a sand tool, no other action can be performed during that round. (5) During the activity, group members can choose to drop in for one round. (6) The participant who takes the last action during a sandplay work is given one chance to make modifications and adjustments to the work but is not allowed to add any sand tools. (7) After a work is completed, the group participants share their intentions during sand tool placement and their feelings during the process. Finally, they choose a name for the work.
